# Hepatosplenic abscesses in an immunocompetent child with cat-scratch disease from Peru

**DOI:** 10.1186/s12941-019-0322-0

**Published:** 2019-07-15

**Authors:** Alexis Alfaro del Pozo, Michelle Angulo-Cruzado, Ricardo Amenero-Vega, Alexander Álvarez-Lulichac, Hugo Fernández-Cosavalente, Joshuan Barboza-Meca, Alfonso J. Rodriguez-Morales

**Affiliations:** 1 Faculty of Human Medicine, Universidad Nacional de Trujillo, Trujillo, Peru; 2 Sociedad Cientifica de Estudiantes de Medicina, Universidad Nacional de Trujillo, Trujillo, Peru; 3 Department of Pediatrics, Faculty of Human Medicine, Universidad Nacional de Trujillo, Trujillo, Peru; 4 Department of Pediatrics, Hospital Belen de Trujillo, Trujillo, Peru; 5 Universidad San Ignacio de Loyola, Lima, Peru; 6 Public Health and Infection Research Group, Faculty of Health Sciences, Universidad Tecnologica de Pereira, Pereira, Risaralda Colombia; 7 Universidad Privada Franz Tamayo/UniFranz, Cochabamba, Bolivia

**Keywords:** Cat-scratch disease, *Bartonella henselae*, Child, Hepatosplenic abscesses, Peru

## Abstract

**Background:**

Cat-scratch disease (CSD) is a zoonotic infection caused by *Bartonella henselae* and *B. clarridgeiae*. The typical manifestations of CSD include regional lymphadenitis and fever. However, CSD can have a wide variety of clinical manifestations that can lead to incorrect diagnoses and prolonged hospital stays.

**Case presentation:**

We present a case of a 3-year-old boy admitted to the pediatric service due to prolonged fever and abdominal pain. He received empirical antimicrobial treatment due to suspicion of infection. Abdominal ultrasound showed hepatosplenic abscesses. An IFA detected the presence of IgG antibodies against *B. henselae* (1:256). Patient was successfully treated with azithromycin and discharged after 7 weeks.

**Conclusions:**

Hepatosplenic abscesses in CSD are rarely reported, particularly in immunocompetent children, with this, only 36 cases in PubMed, Web of Sciences and Scopus bibliographical databases. High rate of suspicion and serological tests availability are of utmost importance in order to detect it and treat it successfully and promptly.

## Background

Cat-scratch disease (CSD) is an infectious zoonotic disease, usually benign, caused by *Bartonella henselae* and *B. clarridgeiae* [1]. The causative agents are gram-negative coccobacilli whose biological cycles involve an intermediate host (often fleas), that maintain transmission between cats [2]. The infected saliva and nails of cats are the main routes of transmission to the human. After inoculation, the bacteria colonize endothelial cells, and then they are released into the bloodstream, where they infect the erythrocytes helped through their virulence factors, such as deformin and motilin that allow the membrane penetration of the red blood cells. Antiapoptotic substances that inhibit the erythrocytic phagosome are also involved, allowing the bacteria to divide and multiply until reaching the critical density that led to clinical disease [3].

The typical form of CSD represents around 90% of cases. This is characterized by the appearance of self-limiting regional lymphadenopathy, accompanied by rash, and fever. Atypical forms would include myocarditis, endocarditis, osteomyelitis, granulomatous conjunctivitis, encephalomeningitis and the Parinaud oculoglandular syndrome, among others [4]. Liver and splenic lesions are considered a rare form of disease (occurring in less than 10% of the cases). Liver abscesses are usually unique but can sometimes be multiple and small (< 2 cm), called micro-abscesses [5, 6]. Abscess of the spleen represents a rare CSD clinical form [7, 8].

The people generally affected by CSD are children and young adults, having an uncertain incidence and little known even in developed countries [9]. The diagnosis is still complicated. However, the serological tests for *Bartonella henselae* using enzyme immunoassay (EIA) or indirect fluorescence assay (IFA) has shown excellent results. Also, the biopsy can be used for granulomatous observation of the infection or using Warthin–Starry stain [10].

Antimicrobial drugs such as erythromycin, gentamicin, quinolones, doxycycline, azithromycin and trimethoprim/sulfamethoxazole (TMP/SMX) have shown favorable results for its treatment. Albeit of that, many of the cases show a spontaneous resolution [11]. We present a case of CSD in an immunocompetent child from Peru, that developed hepatosplenic abscesses.

## Case presentation

A 3-year-old boy from an urban area of Trujillo, Peru, with no completed vaccines and mild anemia, was admitted to our hospital on March 19, 2018, for persistent fever. The mother indicated that her son has been scratched by a stray cat on the anterior region of the left arm with no apparent signs of inflammation.

Eighteen days before admission, he had a fever of 38.5 °C. The day after, colicky abdominal pain begun. He was brought to the emergency ward of another hospital where metamizole was administrated and hours later he was discharged.

Sixteen days before admission, in a private consultation, it was diagnosed apparently with a food poisoning, and he was treated again with metamizole. At this point the fever ceased. Fifteen days before admission, the mother indicated that he had an episode of diarrhea, without mucus or blood, also presenting nausea.

Thirteen days before admission, the fever appeared again, now accompanied with non-productive cough. In a private consultation he was diagnosed with an acute respiratory infection, and was treated with amoxicillin–clavulanic acid (75 mg/kg/day) until his hospitalization, without significant improvement.

The day of the admission to our hospital, his physical exam revealed fever (38.5 °C), pallor (+/+++), a no congestive pharynx and soft and depressible abdomen. His vital signs include a respiratory rate of 24 breaths/min, a cardiac rate of 106 beats/min, an SO_2_ of 96% and a FiO_2_ of 21%. No signs of regional lymphadenitis were observed. At this point, fever of unknown origin (FUO) was suspected. Laboratory tests at income revealed mild anemia, mild thrombocytosis, prolonged coagulation times, including increase fibrinogen and an increased C-Reactive Protein (Table 1).

**Table 1:** Admission laboratory tests

Tests	Results	Reference values
Complete blood count		
Hemoglobin	10.7 g/dL	≥ 11 g/dL
Leukocytes	8.19 × 10^3^ (B: 3, S: 53, N: 56%)	5.5–15.5 × 10^3^ (N: 25–57%)
Platelets	413 × 10^3^	150–400 × 10^3^
Coagulation profile		
PT	13.6 s	10.6–11.4 s
INR	1.24	0.8–1.2
Fibrinogen	470 mg/dL	170–400 mg/dL
PTT	42.9	24–36 s
Urinalysis		
Leukocytes	1–3 per field	0–4 per field
Germs	Negative	Negative
Transaminases		
GOT	31 U/L	10 a 40 U/L
GTP	19 U/L	10 a 34 U/L
CRP	66 mg/dL	< 1 mg/dL

*B* bands, *S* segmented, *N* neutrophils, *PT* prothrombin time, *INR* international normalized ratio, *PTT* partial thromboplastin time, *GOT* serum glutamic oxaloacetic transaminase or aspartate transaminase (AST), *GPT* serum glutamic pyruvic transaminase or alanine aminotransferase (ALT), *CRP* C-reactive protein

**Fig. 1:**
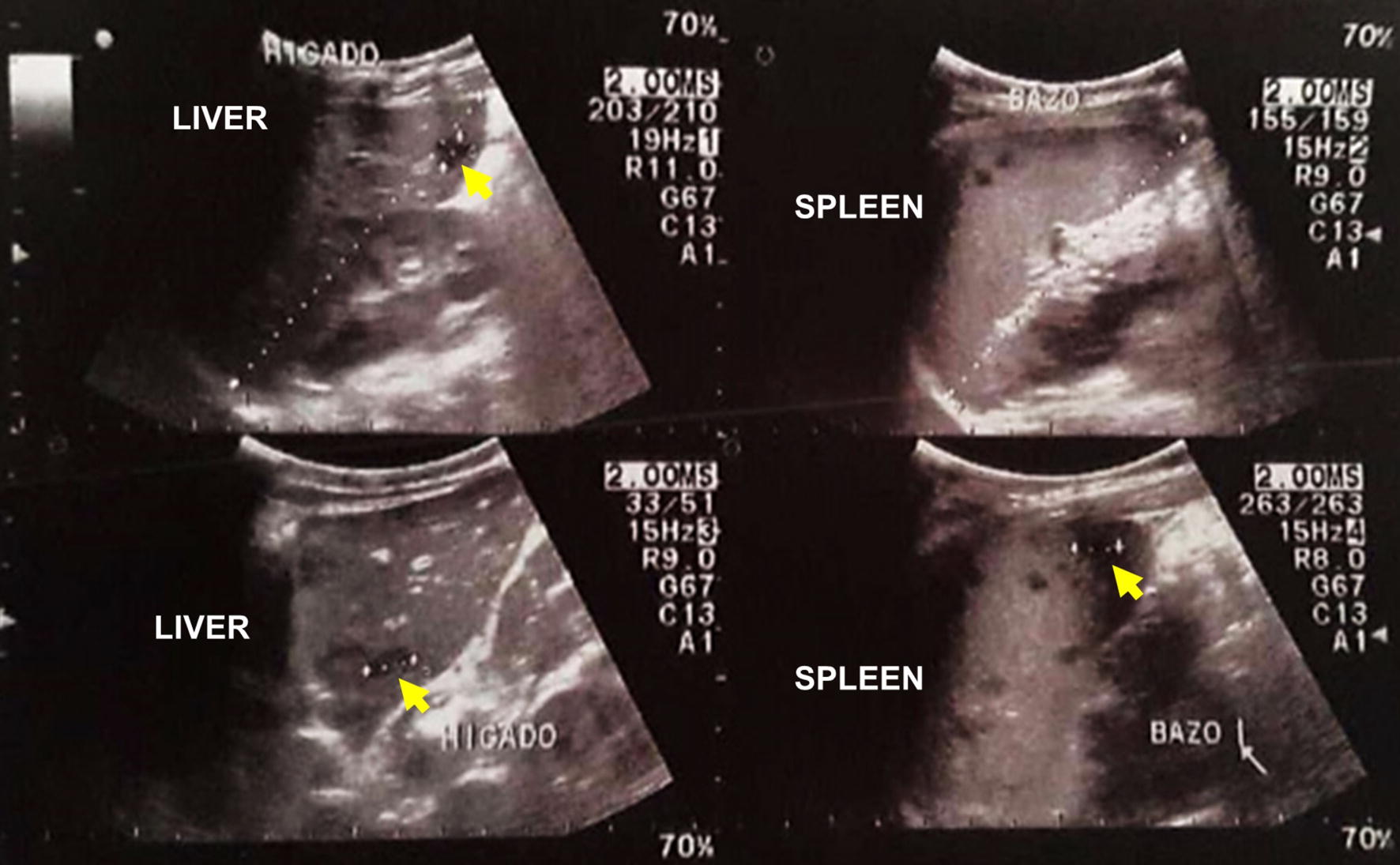
Abdominal ultrasound showing multiple hypoechoic areas with poorly defined edges of up to 11 mm in the liver and in the spleen (yellow arrows indicate the lesions)

The first day of hospitalization, he presented microscopic hematuria, abdominal pain, and persistent fever. An abdominal ultrasound revealed mild hepatomegaly with multiple hypoechogenic formations with non-defined borders < 11 mm at liver, as well as also at spleen, of < 10 mm in the spleen, suggesting hepatosplenic micro-abscesses (Fig. 1).

At day four, treatment with ceftriaxone (81 mg/kg/day) and metronidazole (30 mg/kg/day) was started. On the seventh day of hospitalization, ceftriaxone was changed to imipenem (75 mg/kg/day). Although that, fever persisted for 3 days. Additional laboratory tests at this moment included blood culture for bacteria and fungi, STORCH serologies (VDRL, FTA-Abs, toxoplasmosis, rubella, CMV and EBV, HSV-1, HSV-2), ELISA for HIV, PPD, acid fast bacilli (AFB) from sputum, as well as agglutination tests for *Bartonella bacilliformis* and *Salmonella*. All these tests were negative. An indirect immunofluorescence antibodies (IFA) assay tested positive for IgG against *B. henselae* (titers 1:256) confirming the diagnosis of CSD. Therapy with imipenem and metronidazole was stopped and treatment with azithromycin (10 mg/kg/day) was initiated. One day later fever ceased.

**Fig. 2:**
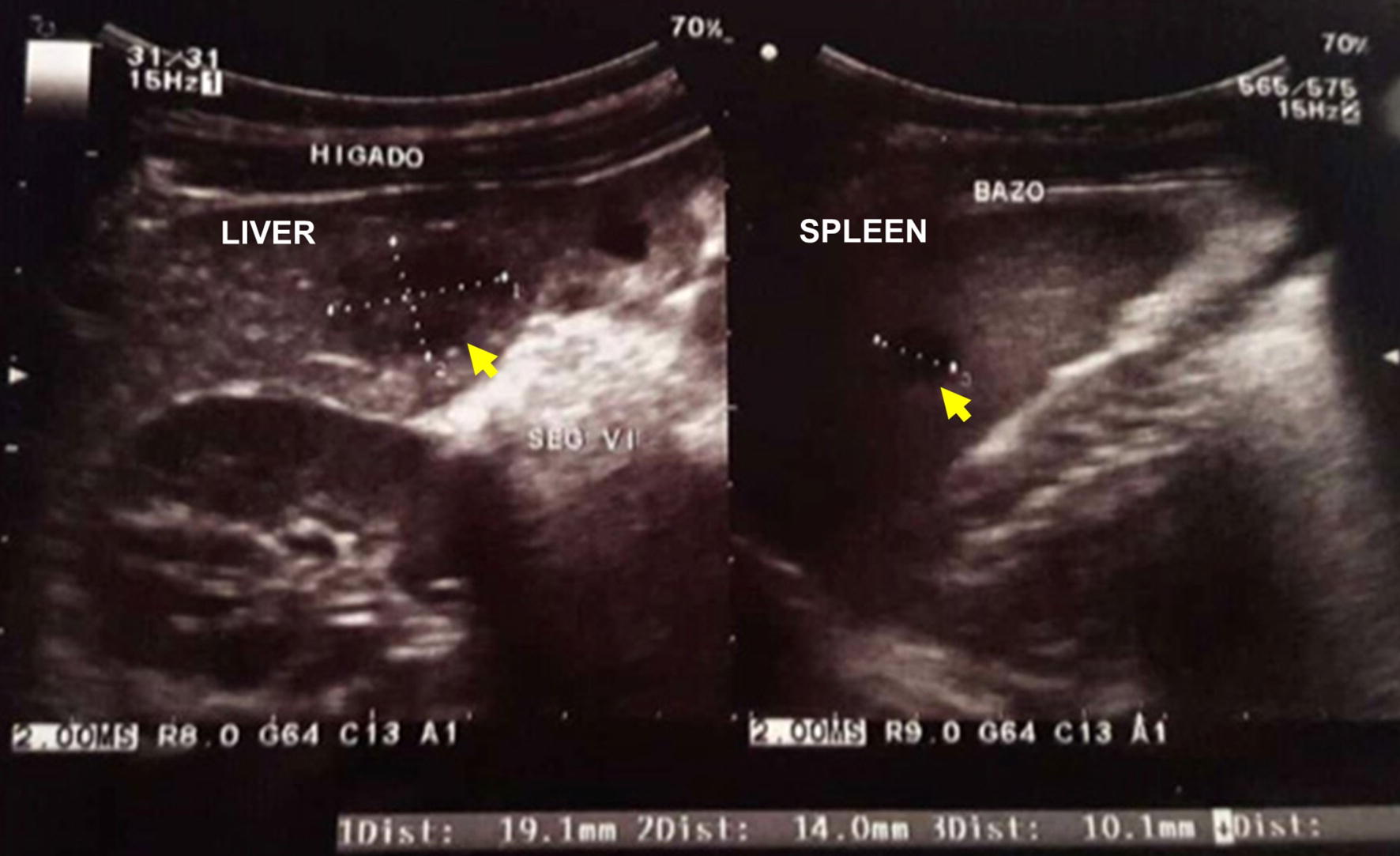
Follow-up ultrasound showing hepatic lesions increased up to 19.1 mm in segments IV and II. Spleen lesions persisted (10.1 mm of diameter) (yellow arrows indicate the lesions)

On the tenth day, an abdominal CT-scan with contrast showed irregular hypovascular nodules of 4.8, 4.6 and 6.5 mm in the liver, in segments II, III and VI, and spleen till 10 mm, confirming the ultrasound findings of micro-abscesses. On the day fifteen, a follow-up ultrasound showed augmented hypoechogenic images in the liver up to 19 mm in segments IV and II of the liver, and up to 10 mm in the spleen (Fig. 2). Two weeks later an additional follow-up ultrasound showed a significant decrease on size of the micro-abscesses.

Then, 2 weeks after finished the antimicrobial therapy and 50 hospitalization days, the patient was discharged. Follow-up till 8 weeks after discharge show no further related alterations.

## Discussion

In CSD, the age-groups more frequently affected are children and adolescents [12]. Although in immunocompetent patients, regional lymphadenitis and fever are common findings, our patient did not present the first [13]. Atypical forms of CSD can have a variety of manifestations including systemic compromise, myocarditis, endocarditis, osteomyelitis and encephalomeningitis, but also hepatosplenic micro-abscesses and are supposed to occur in less than 10% of the cases [14]. Hepatosplenic micro-abscesses are rarely reported in the literature [7, 8, 15–17], especially in children [15, 18]. Although would be considered an old condition [19, 20], current and other recent cases call for keep in mind as a differential diagnosis in adults as well as in children with persistent fever, abdominal pain and lesions at abdominal ultrasound, but particularly on a uncommon clinical presentation. After a comprehensive review of bibliographical databases, PubMed, Web of Sciences and Scopus, we were able to find six previous publications (Table 2). Four of them corresponded to case reports including five cases, and two case series that contain 30 cases, then, summarizing 35 cases previously published cases of immunocompetent children with CSD developing hepatosplenic abscesses (Table 2). The time span of these reports was 17 years, from 1999 to 2016, most of them being from United States (33 out of 35 cases). No previous cases from a Latin American country have been reported. Most patients (22), received rifampin, whilst our case evolved also successfully after treatment with azithromycin. The final outcome of all the reported cases, as well as ours, was cure (Table 2).

**Table 2:** Previous case reports and series of CSD in immunocompetent children with hepatosplenic abscesses

#	Type	N	Year of publication	Age (cases) or age-range (series)	Gender	Location	Clinical presentation	Abscess(es)	Treatment	Outcome	Reference
1	Case reports	2	1996	5-year-old^a^	Male	Houston, TX, USA	Abdominal pain and fever	Hepatosplenic abscesses	Rifampin	Cured	[20]
				4-year-old^a^	Male		Rhinorrhea and fever	Hepatosplenic abscesses	Rifampin	Cured	
2		1	1999	7-year-old	Male	Verona, Italy	Regional lymphoadenitis	Hepatosplenic micro-abscesses	Not reported	Cured	[18]
3		1	2003	3-year-old	Female	Phildelphia, PA, USA	Orbital abscess and osteomyelitis	Hepatosplenic abscesses	Ampicillin–sulbactam plus rifampin	Cured	[36]
4		1	2016	16-month-old	Male	Taoyuan, Taiwan	Disseminated	Hepatosplenic micro-abscesses, resolved after 4 months	Azithromycin	Cured	[15]
5	Case series	11	1997	1.5–13 years-old	Male: 12, female: 7	Atlanta, GA, USA	Fever of unknown origin	Splenic: 11, hepatic: 3	Gentamicin, trimethoprim–sulfamethoxazole, rifampin and ciprofloxacin	Cured	[19]
6		19	1999	2–8 years-old	Male: 5, female: 6	Houston, TX, USA	Fever and abdominal pain	Hepatosplenic: 13, hepatic: 3, splenic: 3	Gentamicin, rifampin, trimethoprim–sulfamethoxazole, ampicillin	Cured	[37]
	Current	1	2019	3-year-old	Male	Trujillo, Peru	Persistent fever	Hepatosplenic micro-abscesses	Azithromycin	Cured	–
	Total	36	1999–2016	16-month–old to 13 year-old	Male: 22, female: 14	USA: 33, Italy: 1, Taiwan: 1, Peru: 1	–	Hepatosplenic: 30, hepatic: 6, splenic: 3	Rifampin: 22, trimethoprim–sulfamethoxazole: 13, gentamicin: 10	All cured	–

^a^These are brothers

Atypical clinical manifestations of CSD would make the diagnosis a difficult task in certain cases, as this is not mostly considered in the differential etiologies. However, history of contact with cats and persistent fever, with serological tests availability help in the clinical and etiological diagnosis [3, 16, 19–21], but given the previous reported cases, evidence of hepatosplenic micro-abscesses would be associated with CSD. Some cases would also develop granulomatous hepatitis with increase in the hepatic transaminases [22]. In our case, patient transaminases remained normal.

The literature points out that clinical criteria such as primary dermal or ocular injury associated with scratching of a cat, presence of local lymphadenopathies close to the scratch and fever are considered in the diagnosis of CSD. But cases, such as ours, can present without ocular or lymphatic alterations.

In addition, laboratory tests that rule out other etiologies should be included. Serological tests, such as the IFA has higher sensitivity and specificity [23]. If possible, tissue biopsies of lymph nodes stained with Warthin–Starry are also helpful in the CSD diagnosis [24]. Serological tests with values higher than 1:64 for IgG and 1:15 for IgM confirm the CSD diagnosis [21].

In recent years, PCR has also proved to be a useful diagnostic tool [25]. Imaging studies are necessary and helpful, especially with abdominal symptoms. CT-scan is the recommended, although ultrasound by an experienced professional has a good diagnostic value and is useful in monitoring, as we did in our case [26].

Appropriate antibiotic treatment of CSD in pediatrics is not well established [27], but azithromycin, as we used, appeared to be the best choice [27]. TMP/SMX may be considered as an alternative antibiotic when azithromycin cannot be used [11, 27].

Unfortunately, routine diagnosis and surveillance of CSD as well as of bartonelloses are not done in Peru and most countries in Latin America, where they are prevalent [28–30]. No previous case of CSD associated with hepatosplenic abscesses nor in children nor in adults was reported before. Even more, CSD and other bartonelloses forms are also neglected in terms of research in the region [29]. Then, surveillance should be established in order to estimate the real prevalence and the real cause of multiple pathologies and their atypical presentations, including FUO, particularly in tropical and subtropical countries [31]. In one of the two case series, CSD was not the initial diagnosis in any of these cases. Five children were referred for evaluation of FUO, but other diagnoses included Kawasaki disease, sinusitis, pyelonephritis, collagen vascular disease, tonsillitis and pharyngitis [19].

Given the high prevalence of infection in cats reported in different studies of CSD [32], and its associated risk as zoonotic disease [33], it is necessary to draw the attention and awareness among the medical community about this differential diagnosis as well as their different clinical presentations and history of contacts with cats, but also with other animals. It is worthy to mention that in addition to cats, also dogs [34], rodents [35] and probably other domestic and peri-domestic animals would be infected with *Bartonella henselae* and should be considered regard the zoonotic risk for humans especially with clinical manifestations.

## Data Availability

Copy of the clinical data of the patient is available.
